# Biased niches – Species response curves and niche attributes from Huisman-Olff-Fresco models change with differing species prevalence and frequency

**DOI:** 10.1371/journal.pone.0183152

**Published:** 2017-08-21

**Authors:** Jana Michaelis, Martin R. Diekmann

**Affiliations:** Vegetation Ecology and Conservation Biology, Institute of Ecology, University of Bremen, Bremen, Germany; University of Fribourg, SWITZERLAND

## Abstract

The study aimed to examine the effects of different numbers of presences and frequencies (proportions) of occurrences of species in a plot data set of forest vegetation on the species response curves and their niche attributes, based on Huisman-Olff-Fresco models (HOF). We modeled responses of 72 to 105 herbaceous forest species along a pH gradient under 14 different random sampling scenarios by varying the number of presences and absences used for model fitting. Mean niche attributes were calculated from 100 repetitive runs for each scenario and species. Re-prediction success of HOF models among the repetitive runs was highest when the total number of plots was high and the frequency of occurrences was low. With low plot numbers and high frequencies, less complicated model types (no response or monotonically increasing/decreasing responses) predominate. Measures of species niche boundaries (limits & borders) and niche width were strongly influenced by changes in sampling characteristics. With an increasing number of presences and an increasing frequency, limits and borders shifted to more extreme values, leading to wider niches. In contrast, species optima showed almost no change between the scenarios. Thus, the detected ecological response of a species often depends on the size of the data set and the relation between presences and absences of a species. In general, high data quantities are required for reliable response curve modeling with HOF models, which prevents the assessment of the responses of many rare species. To avoid undesired bias by differing sampling characteristics when comparing niches between different species or between data sets, the data basis used for model fitting should be adjusted according to the niche attribute in question, for example by keeping the frequency of the species constant.

## Introduction

Understanding species responses along environmental gradients is of great ecological importance and has received much attention by scientists for many years. Analyzing the shape of the response curves is necessary to advance ecological theory [1] and also of practical interest, because some widely used ordination techniques (such as correspondence analysis, CA) are based on the assumption of symmetrical responses [2]. Hence, information about the species responses along gradients is the basis for numerical community analysis and has important implications for continuum theory [3]. Moreover, the shape also determines model attributes, such as optima or niche limits, which can further be used to describe the ecological behavior of species in a simplified manner.

Various methods are available to examine species responses. For example, Gaussian logistic regression, as a special case of generalized linear models (GLMs), allows the modeling of symmetric bell-shaped response curves, which can be used to calculate numerical summaries of the species niche, e.g. the ecological optimum [4]. Gaussian logistic regression has been shown to be a robust technique [5, 6], but the assumption of an unimodal symmetric shape of the niche has sometimes been heavily criticized [7]. Generalized additive models (GAMs) are more flexible in their model shape, but also more difficult to apply and interpret, due to problems with smoother selection and over-fitting [3, 8].

**Fig 1:**
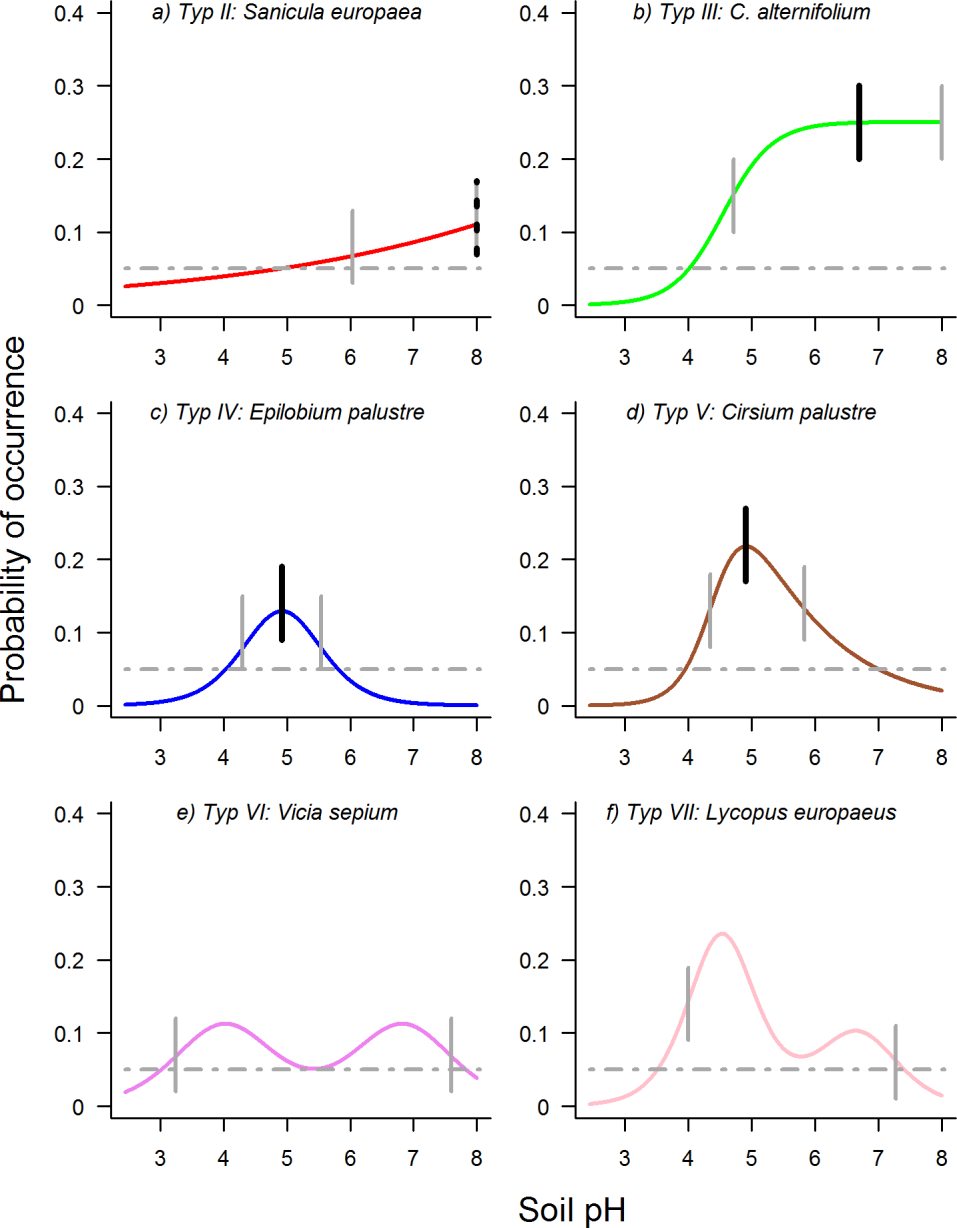
Examples of HOF models (types II-VII) showing the responses of species along a pH gradient. A species with model type I shows no response along the gradient (b shows *Chrysosplenium alternifolium)*. Thick vertical solid lines describe the position of the optima, thin vertical solid lines denote the upper and lower central borders. The dotted grey line corresponds to a probability of occurrence of y = 0.05, and its intersection(s) with the response curve marks the lower and / or upper limit.

Huisman-Olff-Fresco (HOF) models provide a reasonable compromise between statistical correctness, flexibility and ecological interpretability for modeling species responses. They have first been introduced by Huisman et al. [9] as a set of five hierarchical models with an increasing complexity, which were in accordance with ecological theory about species response patterns along gradients. The original paper distinguished the types: no response, increasing or decreasing response with or without plateau as well as skewed and non-skewed unimodal responses (Fig 1A–1D). The original model set-up was implemented as package “gravy” in the R statistical environment by Oksanen and Minchin [3]. This model framework has recently been expanded by Jansen and Oksanen [10] to encompass seven ecological niche patterns, being implemented as the new R package “eHOF”. Apart from the five model types previously mentioned, two bimodal (skewed and symmetric) response shapes were included to cope with species that are restricted to gradient extremes due to competition (Fig 1E and 1F and Table A in S1 File). Although HOF models can take several different shapes, the niche parameters can easily be calculated and used for further analyses. In previous studies, HOF models have been shown to be superior to more restrictive methods (such as GLM or beta functions) and to perform well compared with GAMs that are more flexible [3, 10].

Over the past years, HOF models were widely used in vegetation science and paleoecology, but only rarely in animal ecology. While the main application was to model the responses of single species [11–13], HOF models were also used to analyze changes in species richness [14], species cover [15] or species turnover [16] along different types of gradients. The attributes of the response curve analyzed most often were the shape (in 97% of studies), followed by different kinds of niche boundary measures (limits, borders, thresholds, tolerances; 35%) and the species optimum (32%) (S1 Table).

Despite the frequent use of HOF models, a basic problem of their application is that plot data on species occurrences in combination with environmental measurements in these plots is limited, mainly because the measurement of most environmental factors is time- and cost-intensive. In addition, many species have a low frequency (proportion of presences out of the total number of plots) in data sets even if the overall available number of plots is high, simply because species are often rare in nature for various reasons. Data paucity is of concern, because model fit, results and comparability are influenced by the size and composition of the data set used for modeling. It has been shown that the results of Gaussian logistic regression and of other modeling approaches (incl. GAMs) are heavily affected by the species frequency in the data set and the overall number of data points [17, 18]. However, only 53% of all studies that have used HOF models over the last six years (S1 Table) report the frequencies or the exact number of data points available for each modelled species. Among those, the variability in size and features of the data sets from which models were built is very high: the number of plots varied between 26 and 2691 (median = 207), the minimum number of required species presences ranged from three to 40 (median = 10) and the minimum frequency was within the range of 0.004 to 0.432 (median = 0.04). Moreover, model procedures and evaluations are usually not described in much detail. Thus, it is likely that HOF models are often applied to the data with default package settings and without any prior data adjustment, model stability evaluation or model repetitions. Jansen and Oksanen [10] stated that re-prediction of a unimodal shape works excellent with the “eHOF” package and the stability checks implemented therein, but only if sufficient plot data with an even distribution along the gradient in question is available. Their tests are in line with a suggestion made by Coudun and Gégout [17] for Gaussian logistic regression to use at least 50 occurrences to obtain reliable results. Unfortunately, this pre-condition leads to the exclusion of many less common species from studies, which is why it is rarely followed (S1 Table).

To our knowledge, the only recent study taking into account the potential bias in HOF modeling was done by Reinecke et al. [19] who used an extensive procedure to overcome the problems caused by varying numbers of plots, varying frequencies of species in different regions and uneven sampling along the gradient. In addition, they fitted 50 models, based on random subsets of their data, created an average curve and derived their niche parameters from this average curve, instead of using the parameters provided by the “eHOF” package directly. But their approach still does not take into account between-species differences in data availability, which might lead to differences in model parameters irrespective of the underlying niche features, and thus to biased ecological interpretations.

Given the influence of data paucity and variability on the results of other modeling approaches, we suspect HOF models to be affected by these factors too. This in turn is likely to bias the results, especially if different studies are to be compared or if data sets contain common and rare species at the same time, which is usually the case. To evaluate the impact of the number of presences and species frequency within and between data sets on HOF models and to find a way to correctly apply this method in practical work, we asked the following questions:

- Are the species response curves resulting from HOF models influenced by the different numbers of presences and frequencies?
- If so, does this lead to directional or unpredictable changes in the attributes of the response curves (shape, optimum, limits, niche width), and how extensive are these changes?
- Can we simply apply HOF models to a data set and then compare the niche attributes of rare and common species, or do we have to process our data set differently to get unbiased results?

## Methods

### Data set

Vegetation and soil pH data were compiled from three published vegetation surveys from the geographical region of the Central Upland Range in Germany [20–22]. The data set comprised 1219 plots from all understory community types found in semi-natural deciduous forests in the study region. Soil samples were collected from the upper 10 cm of soil (without litter) and analyzed in different buffer solutions. To be able to compare the different pH values, we converted them all to pH ${}_{CaCl_{2}}$, as described in Michaelis et al. [11]. The soil pH ranges from 2.44 to 8.05 and covers the whole gradient found in the forests of this area. Most plots were obtained from acidic soils whereas the number of sites decreased towards more base-rich conditions (Fig B in S1 File). Plot size varied between 50 and 200 m^2^, depending on the area of homogeneous understory vegetation. For the statistical analysis, we only used herbaceous plants, as they are less directly influenced by forest management compared to trees and because they are supposed to respond to different pH regimes within the upper 10 cm of soil. The data was analyzed as presence/absence.

### Modeling approach

Huisman-Olff-Fresco models were fitted in the R statistical program (v. 3.3.1; R Developmental Core Team [23]) using the package “eHOF” (Jansen and Oksanen [10], version 3.2.2). To improve modeling results even for small data sets, the stability of model choice was double-checked by (1) bootstrapping (100 samplings, default package setting) to ensure model robustness, and (2) the Akaike information criterion corrected for small data sets (AICc, Burnham and Anderson [24], default setting). In case the two procedures differed in their choice for the best model type, the bootstrapping model was preferred. A minimum number of 10 presences and absences in the data set was set as a pre-condition for modeling in the package.

HOF models were applied to 14 different training combinations of presence and frequency constructed by random sampling from the original data set (Table 1). With a constant number of presences, the frequency was changed by varying the number of absences. These are hereinafter referred to as Pre10, Pre25, Pre50, Pre100 and Fre0.068, Fre0.116, Fre0.5, Fre0.714, respectively, and cover a wide range of situations that can be found in ecological studies, from very rare to very common species in small and big data sets. In total 105 species from the original data set met the pre-condition of a minimum of 50 occurrences within the data set to be used in the analysis of scenarios Pre10, Pre25 and Pre50, whereas only 72 species could be used for scenario Pre100 requiring a minimum of 100 occurrences. The scenario Pre100:Fre0.068 could not be modeled due to data shortage of the original data set (1470 plots would have been needed), whereas the scenario Pre10:Fre0.714 did not meet the pre-condition of the “eHOF” package. For the number of presences and frequencies of the species in the original data set, see S2 Table.

**Table 1:** Modeling set-up with 14 different combinations of presence and frequency based on random sampling. Four different presence scenarios (number of randomly selected presences being 10, 25, 50 or 100) combined with four different frequency scenarios (by varying the number of absences) were modeled. Two combinations could not be applied due to model restrictions or data paucity.

Frequency				
Presence	0.068	0.116	0.5	0.714
10	10 / 138	10 / 76	10 / 10	-
25	25 / 345	25 / 190	25 / 25	25 / 10
50	50 / 689	50 / 380	50 / 50	50 / 20
100	-	100 / 762	100 / 100	100 / 40
Presence / Absence				

For each data combination (14) and each species (105/72), model fitting was repeated 100 times, resulting in a total number of 137,100 HOF models. From the 100 repetitions, mean niche parameters, a model stability index and the probability of getting a certain niche parameter were calculated for every species—data combination. Moreover, differences in model choice (which model types were chosen) were evaluated visually.

### Curve/niche parameters

The following model parameters, numerically describing different features of a species niche, were calculated from the HOF models:

1. General species response along the gradient (curve shape)
  The shape of the curve is given by the seven different model types of the HOF approach (Fig 1 and Table A in S1 File). As an estimate of model shape stability, we calculated the *Index of Qualitative Variation* (IQV). This index is zero when all repeated runs arrive at the same model shape, whereas it is one when all model types are chosen equally often [25]. With n being the number of categories (model types) and p being the proportion for each category, the index is calculated as:
  $$IQV=\frac{1-\sum_{i=n}p_{i}^{2}}{\frac{1}{n}*(n-1)}$$
2. Species optimum
  The species optimum describes the highest probability of occurrence of the species along the pH gradient. It can easily be extracted from models of type II, IV and V. In case of model type III (plateau), the optimum is defined as being the midpoint of the plateau. No single optimum can be calculated for the bimodal curve types. Two different optima were distinguished: optimum_any_ and optimum_51_. In case of the optimum_any_, an optimum was assigned to a species whenever an optimum was found in the 100 repetitions, even if this happened only once. For optimum_51_, a value was assigned only if a minimum of 51 repetitions resulted in an optimum.
3. Fixed tolerance limits
  The fixed limits are the points of the curve where the probability of occurrence reaches 0.05. They quantify the statistical limits of occurrence of the species, irrespective of species' fitness or general commonness/rareness in the data set or survey area. The fixed tolerance limits are in the following called 0.05 limits or LowLim and UppLim for the left- and right-hand side of the response curve, respectively. In bimodal models, the outermost 0.05 limits are calculated. We assumed a species to have a LowLim_51_ or UppLim_51_ if at least 51% of model repetitions allowed calculating values. LowLim_any_ and UppLim_any_ were assigned if at least one run gave a corresponding limit.
4. Relative tolerance limits and niche width
  A procedure to calculate relative tolerance limits for each species is already implemented in the “eHOF” package: the central (CB) borders following Heegaard [26]. These are defined as the response values being equal to a specified fraction of the curve maximum: max * e^-0.5^. Similar to the fixed limits, central borders are calculated separately for the left (LowCB) and right (UppCB) hand side of the optimum. Again, the outermost borders are used in bimodal responses. The niche width is defined as the distance between the lower and upper central border.

### Statistical analysis

The statistical analysis was done in R [23]. To evaluate the effects of the varying numbers of presences (Pre) and frequencies (Fre) on the niche parameters (IQV, optima, limits, width), (generalized) linear mixed models ((G)LMM) were used (“lme4” package). Explanatory variables (Pre, Fre) were scaled and centered (for easier interpretation of the results and model fitting except for the IQV analysis). *Species* was set as random intercept to allow between-species variation in pH preference. *Pre* and *Fre* were also included as random slopes, accounting for different responses of single species towards *Pre* and *Fre* changes. Building the model like that allows treating each species as an independent replicate within each of the 14 scenarios (and for each niche parameter), resulting in 105/72 replicates (code example in S3 Table). For niche parameters (e.g. position of the optimum along the gradient), a normal distribution was assumed.Changes in the number of niche parameters (e.g. optima) derived from the repetitive runs were analyzed as count data. In general, these are proportions, because the maximal number of repetitive runs is known (100).In the present case, the binomial models failed to converge and overdispersion was an issue. The analysis as count data with a Poisson distributed model was a compromise and the best approximation, because the results follow the overall trend. Model selection and evaluation followed Zuur et al. [27] and Bolker et al. [28]. P-values for the (G)LMMs were calculated using the *Anova* function from the “car” package.

To see in how far niche parameters for the species were related between the different scenarios and whether there was a directional or unpredictable change, Spearman correlation matrices were used (package “psych”).

## Results

Optima_51_, LowLims_51_ and UppLims_51_ showed similar trends as their corresponding any-parameters, but their usage reduced the size of the data set by up to 90%. Thus, we decided to only present the results for optima_any_, LowLims_any_ and UppLims_any_. Results of 51-parameters, figures of all parameters and a detailed overview of model outputs can be found in S2 File and S3 Table.

### Choice of model types and their variability

**Fig 2:**
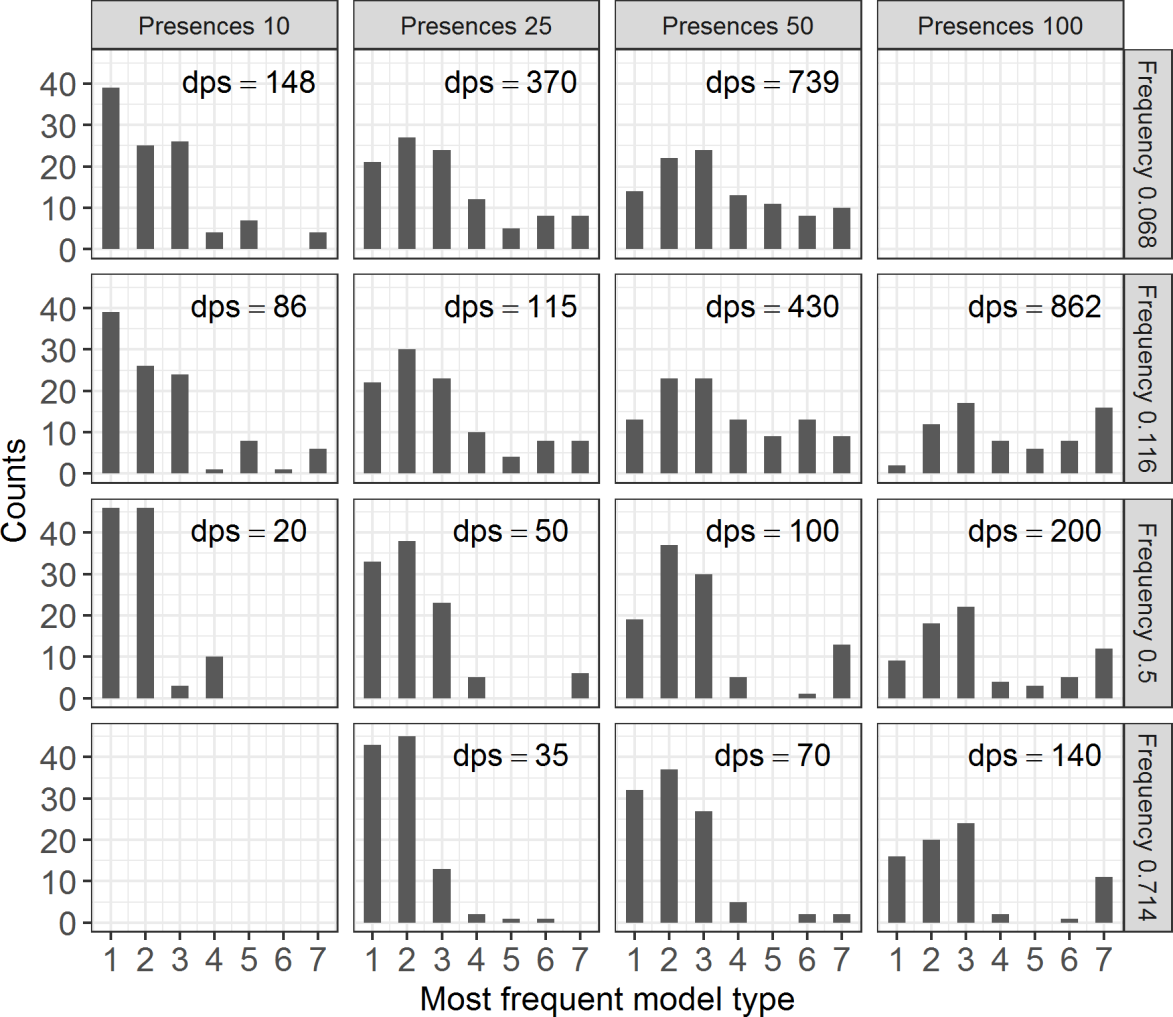
Frequency distribution of model types chosen in the different Pre:Fre scenarios. Dps gives the number of overall data points used for model fitting.

The less complicated model curves of type I and II strongly predominated when the number of presences was low (Pre10) or the frequency was high (Fre0.714). Model choices varied more between the different types in scenarios that include an overall high number of data points and a low frequency (Fig 2).

**Fig 3:**
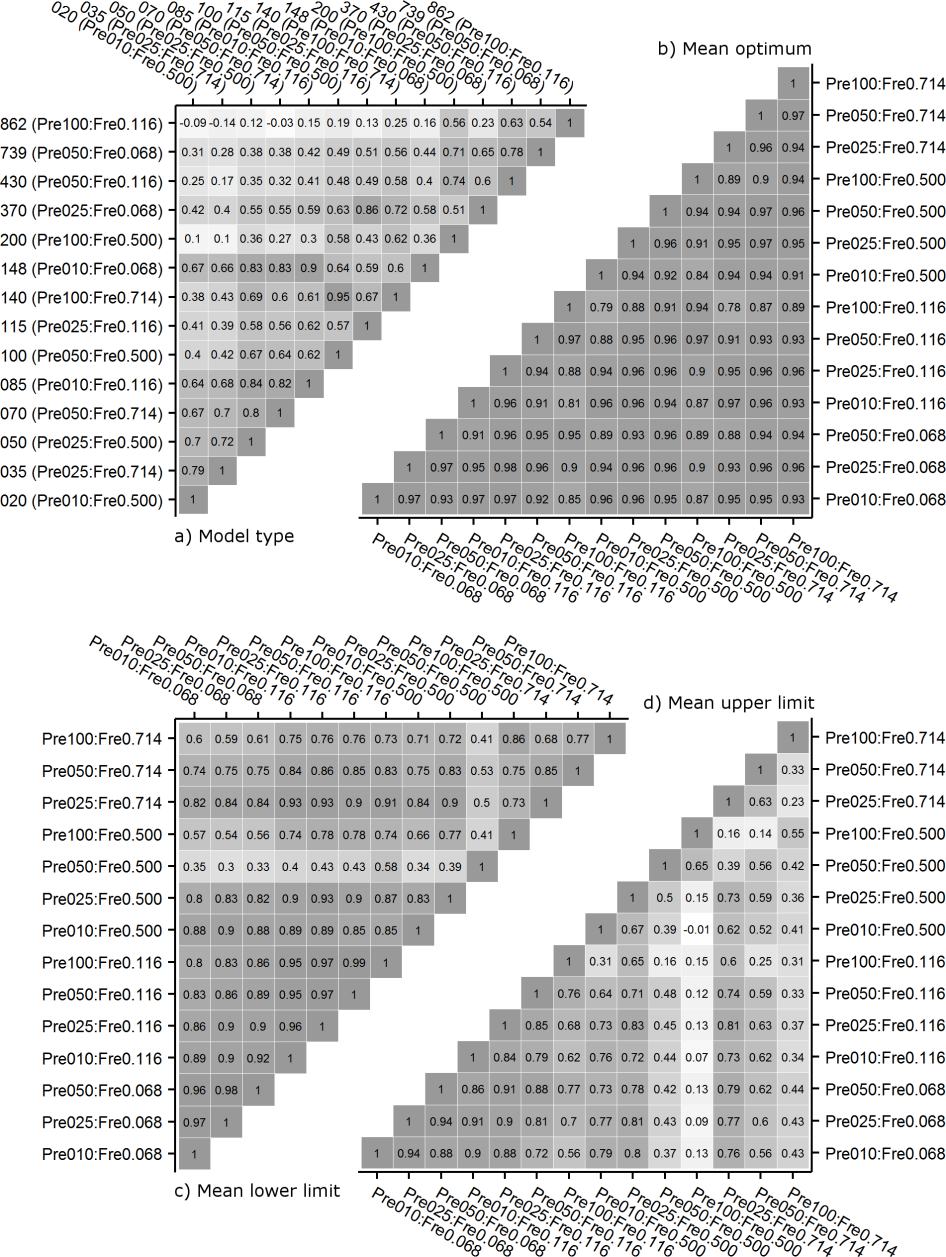
Spearman-correlation matrices for all Pre:Fre scenarios. Given are: a) model type (chosen most often from 100 repetitive model fittings for each species), b) optimum, c) lower limit and d) upper limits. Central numbers show the correlation coefficients. In a) axes are sorted based on the number of data points used for fitting. For b)–d) axes are sorted by frequency and presence numbers.

The pattern of variation in model choice differed between species and between scenarios. Fig 3A shows that there was no correlation for the chosen model type between scenarios when the number of data points differed strongly. This means that aside from the underlying ecological response of species, the chosen model type strongly depended on the size of the data set. This effect can partly be mitigated by equalizing the number of presences or frequencies between data sets.

**Fig 4:**
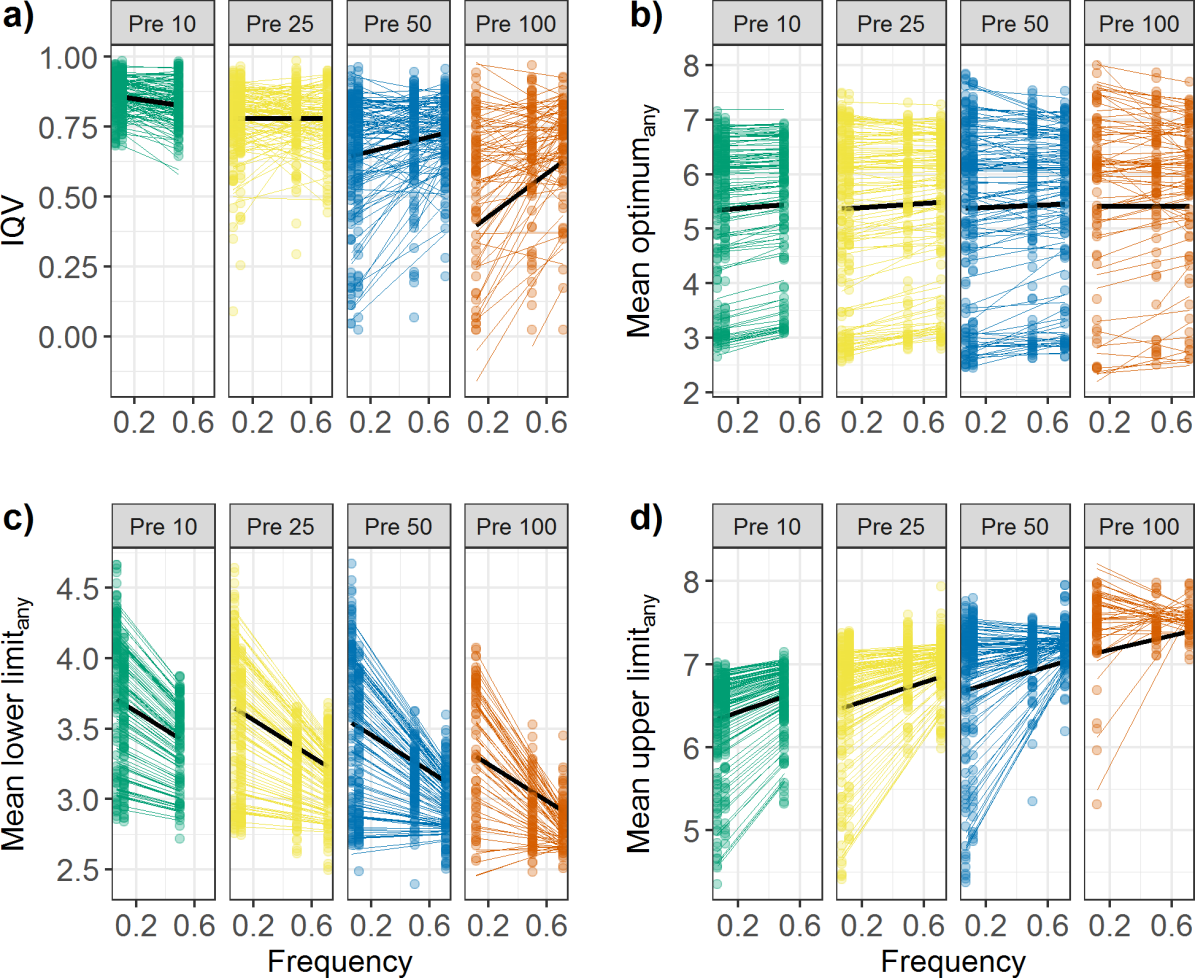
Results of the linear mixed model identifying the trends of niche parameters. Results for a) Index of Qualitative Variation (IQV), b) optimum_any_, c) LowLim_any_ and d) UppLim_any_ along the frequency gradient for all four Pre scenarios are presented. Colored lines show the regressions for every single species, whereas the black line reflects the mean across species (the population trend).

Overall, model variability was very high with IQV being 0.7 on average (Fig 4A). The index significantly decreased with an increasing number of presences (est. = -0.006, p < 0.001). In the Pre10 scenario, the variability decreased with increasing frequency, i.e., the chances to re-predict a certain model type became higher with an increasing ratio of presences to absences. In the Pre25, there was no change in the IQV with changing frequency, whereas in Pre50 and Pre100 the variability showed a strong increase with increasing frequency. The interaction between Pre and Fre was highly significant (est. = 0.005, p < 0.001). In the high presence scenarios (Pre50, Pre100) the overall variability was lower than in the low presence scenarios. The best results in terms of the lowest variability (IQV 0.4) were obtained in the Pre100:Fre0.068 combination, which is also representing the scenario with the highest number of data points used for model building (862; see Table 1). However, when comparing Pre100:Fre0.714 (very common species present in most plots) with Pre10:Fre0.068 (rare species present in a low proportion of plots) it becomes clear that variability was reduced in the Pre100 even if the number of data points (140 vs. 148) was comparable.

### Optimum

The probability of obtaining an optimum curve (types II, III, IV & V) in 100 repetitive runs across all species was 54%. When the frequency increased from 0.068 to 0.714, the probability increased by 1%, a weak change despite the fact that the trend of getting more optima curves with increasing frequency was significant (est. = 0.022, p < 0.001). This frequency-induced increase was more pronounced with high presence numbers (est. = 0.008, p = 0.024).

The positions of species optima_any_ were not influenced by the number of presences. However, we found a significant influence of frequency (est. = 0.041, p < 0.001) and of the interaction between presence and frequency (est. = -0.033, p = 0.002). Overall, the optima_any_ increased with rising frequency, but this tendency was reversed in the Pre100 scenario. For Pre10 the maximum shift was 0.1 pH units, whereas it was -0.01 pH units for Pre100 (Fig 4B). The variation between species increased with an increasing number of presences and extended towards the extremes of the pH gradient. In all scenarios, the optima_any_ positions were closely positively correlated between the different scenarios, with correlation coefficients ranging from 0.79 to 0.97, with a trend towards weaker relationships between the Pre100 scenarios and all others (Fig 3B).

### Lower 0.05 limits and central borders (acidic side of the gradient)

The chances of estimating a lower limit were heavily influenced by frequency (est. = -0.618, p < 0.001) and to a smaller extent by the number of presences (est. = 0.077, p < 0.001). While an increase in presences from 10 to 100 led to a 5% higher probability to detect a limit at low frequency levels, an increase in frequency otherwise led to a reduction in the chance of observing a lower limit at the 0.05 level of up to 37%.

The LowLim_any_ of species were strongly influenced by the number of presences (est. = -0.152, p < 0.001) and the frequency (est. = -0.205, p < 0.001) in the data used for model fitting. Across species, the LowLim_any_ shifted to more extreme (low) pH values by 0.4 units with an increasing number of presences and increasing frequency, respectively. This resulted in the highest pH LowLims_any_ in the Pre10:Fre0.068 scenario and the lowest pH LowLims_any_ in the Pre100:Fre0.717 scenario, with a difference of 0.8 pH units (Fig 4C). The higher the frequency, the lower was the variation among the LowLim_any_ values. LowLims_any_ from different scenarios were closely correlated with an exception being the Pre50:Fre0.5 scenario, which was not or only weakly correlated to all other scenarios (Fig 3C).

Lower central borders also varied depending on the scenario, which is in accordance with the results for LowLims_any_. There was a shift towards more acidic conditions with an increasing number of presences (est. = -0.048, p < 0.001) and an increasing frequency (est. = -0.178, p < 0.001), with a significant interaction effect (est. = -0.032, p < 0.001). Across species, lowest pH borders (pH 3.36) were found for the scenario where species were very common and highly frequent (Pre100:Fre0.714). On the other hand, borders under more base-rich conditions (pH 3.86) were detected for the rarest species scenario (Pre10:Fre0.068). Overall, the maximum shift between these scenarios was less pronounced than for the lower limits, indicating that the lower borders were less influenced by differences in the data sets than the lower limits. This was confirmed by the strong correlations between scenarios (coefficients 0.75–0.99).

### Upper 0.05 limits and central borders (base-rich side of the gradient)

The number of species with detectable ecological limits towards high pH values was significantly reduced with high presence numbers (est. = -0.354, p < 0.001) and high frequencies (est. = -0.879, p < 0.001), with frequency being more influential. For example, the most contrasting scenarios Pre10:Fre0.068 and Pre100:Fre0.714 differed by 33 species that had a limit in the first, but not in the second scenario.

The position of the UppLim_any_ along the pH gradient was shifted to more base-rich values with increasing presence (est. = 0.292, p < 0.001) and increasing frequency (est. = 0.173, p < 0.001). For many species, this resulted in UppLims_any_ predicted to be outside of the pH gradient measured in this study, and thus the number of species having an UppLim_any_ decreased. A mean increase of 0.4 pH units was found along the frequency gradient for Pre25. Changing the number of presences from 25 to 100 in Fre0.714 led to an increase in UppLim_any_ by 0.5 pH (Fig 4D). The correlations between the UppLims_any_ of different scenarios were very high among the low frequency settings. In the high frequency scenarios, the correlation coefficients were much lower, caused by stronger and less systematic changes in UppLim_any_ values and a smaller data set. This was especially true for the Pre100:Fre0.5 case (Fig 3D).

Again, the borders showed the same trend as the limits, but less distinct. They shifted to higher values with species getting more common, both regarding the number of presences (est. = 0.158, p < 0.001) and frequency (est. = 0.044, p < 0.001), with Pre being the main driver. Maximal pH shift across all species was 0.5 pH units. The correlation matrix between the Pre100 scenarios and other scenarios was relatively weak, but in general all coefficients were high (0.6–0.97).

### Niche width

Given that both lower and upper central borders shifted towards the extremes with an increasing number of presences and an increasing frequency in the data set, it is not surprising that species niches got wider when both parameters increased (*Pre*: est. = 0.238, p < 0.001; *Fre*: est. = 0.193, p < 0.001). Thus, irrespective of their ecological preferences or their occurrence in the original (natural) data set, species had wider niches when they were better presented in the data set used for model fitting. Mean niches spanned a range of 3.2 pH units and could shift, depending on the scenario, up to 0.5 units. The main variation between scenarios was found between low and high frequency scenarios in the correlation matrix, but, as found for the central borders, relationships were in general quite strong (coef. 0.63–0.98).

## Discussion

The Huisman-Olff-Fresco models are clearly influenced by the number of presences and the frequency of species in a data set. Response curves and their attributes differed significantly between the tested scenarios, irrespective of the underlying (“true”) ecological behavior of the species, despite the fact that several model stability mechanisms were used (bootstrapping, cAIC, repetitive random subsampling and averaging of results). However, the impact and effect size of changes in species frequency and presence differed between the niche attributes derived from the models. Thus, it is obvious that HOF model results cannot simply be compared between rare and common species, differing in presence and frequency, or between data sets of different size. To assess the extent of this possible bias and propose a solution, it is necessary to examine each model parameter separately.

Most previous studies using HOF models analyzed the shape of the species response curve along the gradient, i.e. the model type. The re-prediction success in model types was very low. Even for the—in terms of the total number of plots—largest data scenario (Pre100:Fre0.116) the mean Index of Variation across species was still 0.4 (for a visual impression of this issue see the animations in S3, S4 and S5 Files). This result, however, overestimates the actual differences between the curves, because the IQV takes the different model types as being completely different categories. Visual inspection of the curves revealed that these, in many cases, differed only slightly, although different model types were chosen: bimodal models with an extremely flat second hump, for example, are very similar to unimodal curves. These results confirm the findings by Jansen and Oksanen [10] that re-prediction rates rapidly decrease in small data sets and with uneven sampling distribution. Mohler [29] suggested to sample more extensively at the gradient extremes to increase prediction success in Gaussian regression, and thus to intentionally use an unbalanced data set. In the present data set, the acidic extreme of the gradient was sampled intensively, while the more base-rich part of the gradient was sampled less well. Despite the high sampling intensity at low pH, curve shape and also lower limits and lower central borders varied strongly. When models are fitted with few data points or high frequencies, usually curves of type I to III are selected. Models that are more complicated can only be found with a high total number of plots in combination with low frequencies. This result is expectable, because the AICc penalizes, apart from the addition of parameters to the model, small sample size. Using the Bayesian Information Criterion (BIC) instead, adds an even stronger penalty for small sample size, which results in a stronger restriction in model choice and in the selection of simple models in all data scenarios (data can be found in S6 File). This further adds to the observation that results obtained with small data sets are not reliable. In general, accuracy of response or distribution models usually improves with additional information, but plateaus exist [30]. The most rapid improvement of model accuracy in logistic regression was found to take place in scenarios with less than 20 presence points in the study by Stockwell and Peterson [30]. We also found an increased variety in the frequency distribution of model types between ten and 25 presences in the present data (Fig 2), but the within repetition variation, i.e. the IQV, did not improve much. Hence, our results support the findings that a minimum of 50 occurrences is necessary to obtain reliable re-prediction results and maximal model accuracy [17, 30]. In addition, our results suggest that a low frequency of occurrence in the data set is important as well. In general, a high re-prediction success of curve shapes (or model types) can be achieved only with a high total number of plots, which are evenly distributed along the whole gradient, and free choice of the model types by a low frequency of the single species in this data set, regardless of which IC is used. In case between-species differences are to be compared, the frequency should be kept constant for all species, regardless of their natural occurrence rates.

The optima_all_ of species were less influenced by differing numbers of presences or frequencies than all other niche attributes. Although significant differences between the scenarios were found, the actual effect sizes were very small. For example, differences of up to 0.1 pH units are within the range of variation that can be expected when combining data from different sources, with samples being measured in different labs or solvents. Thus, we do not see the need for any data manipulation when considering optima, neither regarding number of presences nor regarding frequency.

The fixed 0.05 limits_any_ were very sensitive to changes in the number of presences and in frequency. An increase in the number of presences led to a shift to more extreme values on the left- and right-hand side of the gradient, respectively, and to fewer species having their limits within the gradient range. An increase in frequency, in general, resulted in more extreme limits and a high chance for the response curve to no longer cross the 0.05 line. However, it also caused a strong clustering of limits at a certain pH level, which induces a shift to less extreme limits for some species, causing weaker correlations between the scenarios and making the effects of data differences on single species less predictable. The clustering was probably caused by the fact that, in high frequency scenarios, most species showed responses of type I–III with a very high probability of occurrence along the gradient and very steep slopes towards the gradient ends. To be able to compare fixed limits between species or data sets, we therefore suggest to use low frequencies or to enlarge the gradient by additional sampling to allow for smooth slopes, variation of limit positions and to avoid clustering. Moreover, it is necessary to keep both presence numbers and frequency constant.

The central borders_any_ were somewhat less influenced by manipulations of the data set than the limits. They also shifted to more extreme values with increasing number of presences and frequency, and suffered from the same clustering effect. The niche width suffered from the shift of the lower central borders to more acidic conditions and from the shift of upper borders to more base-rich conditions. This additive effect led to a high sensitivity of the niche width towards changes of presences and frequencies. Thus, we suggest applying occurrence data with a low frequency and keeping frequency and number of presences constant when comparing different species with each other or across data sets.

In the present study, curve parameters were derived by calculating mean parameter values from 100 repetitive model runs with random sampling. Optima_any_ and limits_any_ were heavily influenced by unexpected outlier curves, resulting in misleading optima or limit values. For example, it is likely that a species is not facing its limit within the gradient range when 99 curves give no limitand a limit is assigned in only one repetition. It thus seems to be more reasonable to use the optima_51_ and limits_51_, but this reduced the present data set by up to 90% and led to similar results. An alternative approach is applied by Reinecke et al. [19] who created an average curve from repetitive runs and derived their parameters from it. In how far and to what extent these parameters are influenced by the number of presences and frequency remains to be tested.

Based on our results, it appears to be difficult to establish the "true" niche characteristics of species, because all model attributes, apart from the optimum, are heavily influenced by the properties of the data set. In practice, we believe that it is necessary to adjust a data set in terms of species presences and frequencies to compare niches between species or between different data sets, according to the above-mentioned criteria. Of course, this data manipulation has a strong impact on the results, i.e. the absolute values, but at least the effects then are consistent within the study. In general, a high number of presences combined with an overall low frequency seems to be the best option to obtain good results from HOF models. In the present data set, only 30% of the plant species met the conditions of having 50 occurrences or more, hence 70% of the species could not be modeled. These restrictions caused by data paucity are not unique to vegetation science, but can also be found in animals. For example, in Mexican bird populations, the median of available data points was 83 for all species with a heavy right skewed frequency distribution [31]. The high requirements with respect to the properties of the data set for successfully modeling species response curves and the fact that few species meet these requirements, even in big data collections, stress the need for continued plot-based sampling of species and associated environmental data, and for further effort to make existing data accessible to the scientific community. Moreover, detailed information on the data used for response modeling should be given in future studies in order to enable comparisons across data sets and for meta-analyses.

## Supporting information

S1 File HOF model formulas and distribution of pH measurements along the gradient. (DOCX)

S2 File Figures of niche parameters that are not given in the article. (DOCX)

S3 File Description for appendices S4 File and S5 File. (DOCX)

S4 File Animation of HOF models with increasing presence numbers. (GIF)

S5 File Animation of HOF models with increasing frequency. (GIF)

S6 File Comparison between AIC, AICc and BIC for model selection (DOCX)

S1 Table Literature survey. (XLSX)

S2 Table Species data: Original data attributes and meta data of Pre:Fre scenarios. (XLSX)

S3 Table Results of (generalized) linear mixed models. (XLSX)

S4 Table Vegetation data used for modelling. (XLSX)
