# Supplementary material for: Biased niches – Species response curves and niche attributes from Huisman-Olff-Fresco models change with differing species prevalence and frequency
Source: PLoS One. 2017 Aug 21;12(8):e0183152. doi: 10.1371/journal.pone.0183152 (PMC5565184; doi:10.1371/journal.pone.0183152)
Supplement: S1 File — (DOCX) [file pone.0183152.s001.docx]

**Appendix S1 File**

Table A. Model formulas for the seven different model types (modified from Jansen and Oksanen (2013)). Data were analyzed as presence/absence, hence M=1.

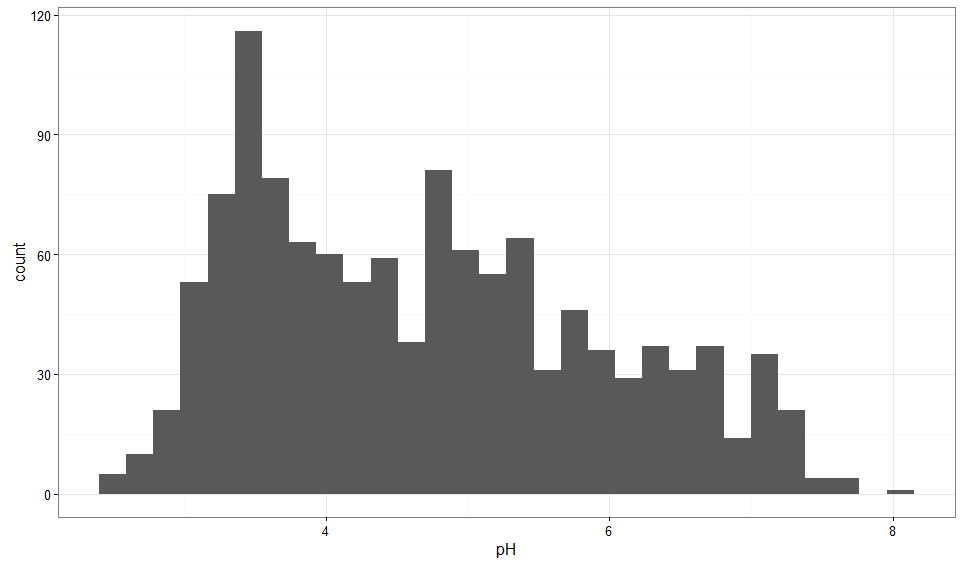


Fig B. Frequency distribution of soil pH measurements in 1219 plots of semi-natural deciduous forest in the Central Upland Range in Germany.
