# Supplementary material for: Biased niches – Species response curves and niche attributes from Huisman-Olff-Fresco models change with differing species prevalence and frequency
Source: PLoS One. 2017 Aug 21;12(8):e0183152. doi: 10.1371/journal.pone.0183152 (PMC5565184; doi:10.1371/journal.pone.0183152)
Supplement: S2 File — (DOCX) [file pone.0183152.s002.docx]

**Appendix S2 File**: Description of results for 51-parameters and visualized results of (generalized) linear mixed models and correlation matrices for all parameters that are not shown in the article.

Optimum_51_

The mean optima_51_ showed similar patterns as described above, but were significantly influenced by presence, frequency and their interaction. On average, the optimum_51_ decreased by 0.02 pH units with increasing number of presences (est. = -0.026, p = 0.039) and increased by 0.02 units with increasing frequency (est. = 0.028, p < 0.001), whereas this effect again leveled off with increasing numbers of presences (est. = -0.018, p = 0.004). Correlations between the different scenarios were even stronger than before (coefficients 0.87 to 0.99).

LowLims_51_

Although the number of observations was strongly reduced in the LowLims_51_, the same significant relationships between *Pre* (est. = -0.004, p > 0.001), *Fre* (est. = -1.313, p < 0.001) and the limits could be found as described above. The shift of the limits_51_ towards lower pH values was even more pronounced, with a maximum difference of pH 1.1 units between the highest and the lowest estimated limit_51_. Because the number of species having a LowLim_51_ in the high frequency scenarios was reduced, only few significant correlations could be found for these scenarios, even if the correlation coefficient was high in some cases. Still, the correlations between scenarios with similar frequencies, compared to strongly differing frequencies, were much higher.

UppLims_51_

Using UppLims_51_ reduced the data set by 90%. Still, species UppLims_51_ shifted to more base-rich soils under high presence numbers (est. = 0.169, p < 0.001) and a high frequency (est. = 1.223, p < 0.001). Both parameters significantly influenced the UppLims_51_, as described for the UppLims_any_, but the impact of frequency changes was even stronger. An increase in frequency in the Pre25 resulted in a shift of the UppLims_51_ by as much as 2.5 pH units. The correlations between scenarios showed the same trend as mentioned for the UppLims_any_, but with Pre25:Fre0.5 having unexpectedly low correlation coefficients.

**Optimum_51_**

Fig A. Results of linear mixed models identifying the trends for the optimum_51_ along the frequency gradient for all four presence scenarios. Colored lines show the regressions for the single species, whereas the black line shows the overall trend across species (population trend).

Fig B. Spearman - correlation matrix for the mean optimum_51_ showing all possible combinations of scenarios. Axes are sorted by frequency and number of presences. Given are the correlation coefficients.

**Lower limits_51_**

Fig C. Results of linear mixed models identifying the trends for the lower limits_51_ along the frequency gradient for all four presence scenarios. Colored lines show the regressions for the single species, whereas the black line shows the overall trend across species (population trend).

Fig D. Spearman - correlation matrix for the mean lower limits_51_ showing all possible combinations of scenarios. Axes are sorted by frequency and number of presences. Given are the correlation coefficients.

**Upper limits_51_**

Fig E. Results of linear mixed models identifying the trends for the upper limits_51_ along the frequency gradient for all four presence scenarios. Colored lines show the regressions for the single species, whereas the black line shows the overall trend across species (population trend).

Fig F. Spearman - correlation matrix for the mean upper limits_51_ showing all possible combinations of scenarios. Axes are sorted by frequency and number of presences. Given are the correlation coefficients.

**Lower central borders**

Fig G. Results of linear mixed models identifying the trends for the mean lower central border (LowCB) along the frequency gradient for all four presence scenarios. Colored lines show the regressions for the single species, whereas the black line shows the overall trend across species (population trend).

Fig H. Spearman - correlation matrix for the mean lower central border (LowCB) showing all possible combinations of scenarios. Axes are sorted by frequency and number of presences. Given are the correlation coefficients.

**Upper central borders**

Fig I. Results of linear mixed models identifying the trends for the mean upper central border (UppCB) along the frequency gradient for all four presence scenarios. Colored lines show the regressions for the single species, whereas the black line shows the overall trend across species (population trend).

Fig J. Spearman - correlation matrix for the mean upper central border (UppCB) showing all possible combinations of scenarios. Axes are sorted by frequency and number of presences. Given are the correlation coefficients.
