# Supplementary material for: Biased niches – Species response curves and niche attributes from Huisman-Olff-Fresco models change with differing species prevalence and frequency
Source: PLoS One. 2017 Aug 21;12(8):e0183152. doi: 10.1371/journal.pone.0183152 (PMC5565184; doi:10.1371/journal.pone.0183152)
Supplement: S3 File — (DOCX) [file pone.0183152.s003.docx]

**Appendix S3 File**: Description of appendices S4 File and S5 File.

Appendix S4 File: Animation of changing HOF models for *Brachypodium sylvatica* with increasing number of presences and a constant frequency of 0.18. The upper panel shows the increasing number of presences used to fit the model. Below, the resulting curves can be seen, with soil pH on the x-axis and the probability of occurrence on the y-axis. It can be seen that the resulting model type and also model parameters, e.g. optima position, are getting more stable with increasing presence.

Appendix S5 File: Animation of changing HOF models for *Brachypodium sylvatica* with increasing frequency, but a constant number of 50 presences. The upper panel shows the increasing frequency, the lower panel gives the corresponding model. Model shape is quite variable, due to the relatively low number of presences used (see also Appendix 7). Still, the probability of occurrence is strongly increasing, followed by steeper slopes and large differences in e.g. limit positions.
