# Supplementary figures and images for: Biased niches – Species response curves and niche attributes from Huisman-Olff-Fresco models change with differing species prevalence and frequency
Source: PLoS One. 2017 Aug 21;12(8):e0183152. doi: 10.1371/journal.pone.0183152 (PMC5565184; doi:10.1371/journal.pone.0183152)

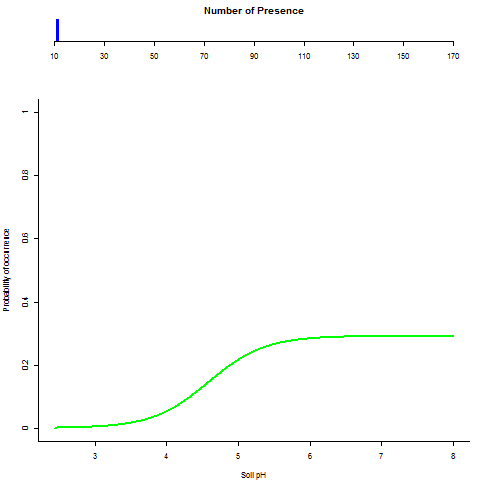

Supplement: S4 File — (GIF) [file pone.0183152.s004.gif]

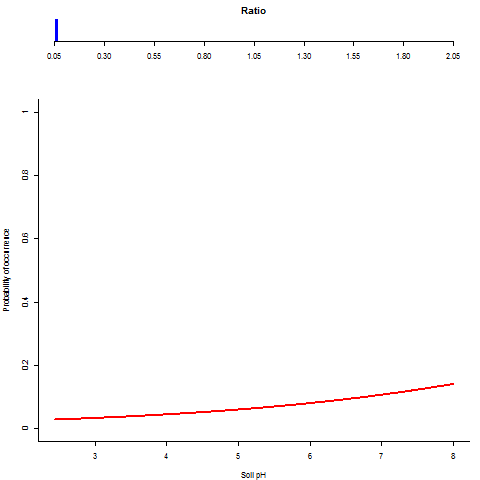

Supplement: S5 File — (GIF) [file pone.0183152.s005.gif]
