# Supplementary material for: Biased niches – Species response curves and niche attributes from Huisman-Olff-Fresco models change with differing species prevalence and frequency
Source: PLoS One. 2017 Aug 21;12(8):e0183152. doi: 10.1371/journal.pone.0183152 (PMC5565184; doi:10.1371/journal.pone.0183152)
Supplement: S6 File — (DOCX) [file pone.0183152.s006.docx]

**Appendix S6 File: Comparison between AIC, AICc and BIC for model selection.**


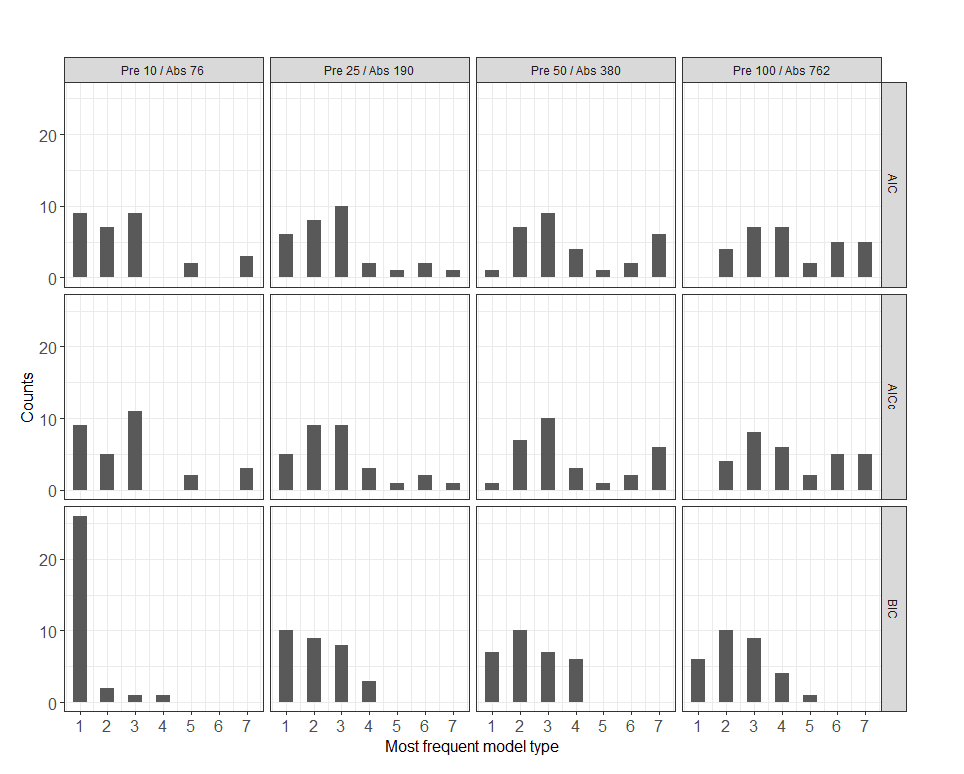


Fig S6 File. Frequency distribution of model types chosen in four exemplary model scenarios with three different information criteria (AIC, AICc and BIC). Thirty species were modelled following the procedure described in the article, but with differing information criteria for model selection.
